# Radiosensitization of HNSCC cells by EGFR inhibition depends on the induction of cell cycle arrests

**DOI:** 10.18632/oncotarget.9161

**Published:** 2016-05-04

**Authors:** Malte Kriegs, Ulla Kasten-Pisula, Britta Riepen, Konstantin Hoffer, Nina Struve, Laura Myllynen, Friederike Braig, Mascha Binder, Thorsten Rieckmann, Reidar Grénman, Cordula Petersen, Ekkehard Dikomey, Kai Rothkamm

**Affiliations:** ^1^ Laboratory of Radiobiology & Experimental Radiooncology, University Medical Center Hamburg – Eppendorf, Hubertus Wald Tumorzentrum – University Cancer Center Hamburg, 20246 Hamburg, Germany; ^2^ Department of Oncology and Hematology, BMT with section Pneumology, University Medical Center Hamburg – Eppendorf, Hubertus Wald Tumorzentrum – University Cancer Center Hamburg, 20246 Hamburg, Germany; ^3^ Department of Otorhinolaryngology and Head and Neck Surgery, University Medical Center Hamburg – Eppendorf, Hubertus Wald Tumorzentrum – University Cancer Center Hamburg, 20246 Hamburg, Germany; ^4^ Department of Otorhinolaryngology – Head and Neck Surgery, University of Turku and Turku University Hospital, 20521 Turku, Finland

**Keywords:** EGFR, HNSCC, targeting, radiosensitization, cell cycle

## Abstract

The increase in cellular radiosensitivity by EGF receptor (EGFR) inhibition has been shown to be attributable to the induction of a G1-arrest in p53-proficient cells. Because EGFR targeting in combination with radiotherapy is used to treat head and neck squamous cell carcinomas (HNSCC) which are predominantly p53 mutated, we tested the effects of EGFR targeting on cellular radiosensitivity, proliferation, apoptosis, DNA repair and cell cycle control using a large panel of HNSCC cell lines. In these experiments EGFR targeting inhibited signal transduction, blocked proliferation and induced radiosensitization but only in some cell lines and only under normal (pre-plating) conditions. This sensitization was not associated with impaired DNA repair (53BP1 foci) or induction of apoptosis. However, it was associated with the induction of a lasting G2-arrest. Both, the radiosensitization and the G2-arrest were abrogated if the cells were re-stimulated (delayed plating) with actually no radiosensitization being detectable in any of the 14 tested cell lines. Therefore we conclude that EGFR targeting can induce a reversible G2 arrest in p53 deficient HNSCC cells, which does not consequently result in a robust cellular radiosensitization. Together with recent animal and clinical studies our data indicate that EGFR inhibition is no effective strategy to increase the radiosensitivity of HNSCC cells.

## INTRODUCTION

For head and neck squamous cell carcinomas (HNSCC) the only approved molecular targeting approach in combination with X-irradiation (IR) is the targeting of the epidermal growth factor receptor (EGFR) using the antibody cetuximab. This is based on the data from Bonner et al. [1] which showed an increased overall survival by about 8% compared to radiotherapy alone when advanced HNSCC patients were treated additionally with cetuximab. It is assumed, that targeting of the EGFR improves tumor control at least in part by increasing the cellular radiosensitivity of the tumor cells [2]. This cellular radiosensitization is thought to result from inhibited DNA double strand repair [3-5] and increased apoptosis [6, 7]. Additionally, many studies reported an inhibition of cell growth and an accumulation of cells in distinct phases of the cell cycle [6–9]. However, cellular radiosensitization by EGFR targeting is still a matter of discussion since some tumor cells show sensitization but others do not [5, 10, 11].

We have recently demonstrated for non-small cell lung carcinoma (NSCLC) cell lines, that EGFR inhibition by the small molecule inhibitor erlotinib induces a cell cycle arrest in G1. This G1 arrest correlated with cellular radiosensitization under pre-plating conditions in a p53-dependent manner. However, the sensitization did not translate into improved tumor control when tumors were treated with fractionated IR [10]. Since re-stimulation by re-plating (delayed plating) abolished this G1 arrest and reversed the sensitization, the failure to improve tumor control might result from re-stimulating events during tumor repopulation in the course of fractionated therapy [12, 13].

Knowing about the importance of cell cycle regulation and culturing conditions in terms of radiosensitization by EGFR targeting, we asked whether EGFR targeting by cetuximab and erlotinib induces cellular radiosensitization of human papilloma virus (HPV)-negative HNSCC cells under different culturing conditions. To facilitate a relevant outcome we included a large panel of 14 different cell lines in this study.

## RESULTS

### Deficient p53 signaling in HNSCC cell lines

**Figure 1:**
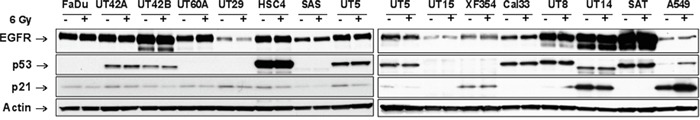
EGFR expression and p53 signaling in HNSCC cell lines Different HNSCC cells were irradiated with 6 Gy. EGFR expression and induction of p53 and p21 were analyzed 4 h later by Western blot using whole cell lysates. A549 cells served as a positive control for p53 and p21 induction and actin as a loading control.

In this study we wanted to determine if EGFR targeting is able to radiosensitize HNSCC cells. Because our previous studies have demonstrated the importance of intact p53 signaling in the context of EGFR targeting, we tested p53 and p21 induction in 14 different HNSCC cell lines using Western blot. In contrast to the p53 wild type (wt) NSCLC cell line A549 none of the HNSCC cells showed p53 or p21 induction 4 h after IR (Figure 1, Table 1). This and the strong basal level of p53 in some cell lines is in agreement with data reporting p53 mutations in 12 of the used HNSCC cell lines (Table 1) [14, 15].

**Table 1:** Cell lines characteristics

Linie	Origin	HPV	p53	p21 induction	EGFR(Exon 19-21)	EGFR amplification	KRAS status (Exon 2-4)
UT-SCC 5	linguae	−	mut*	−	wt**	−**	wt#
UT-SCC 8	larynx	−	mut**	−	wt**	+**	wt#
UT-SCC 14	linguae	−	mut*	−	wt**	+**	wt#
UT-SCC 15	linguae	−	mut*	−	wt**	−**	wt#
UT-SCC 29	larynx	−	mut#	−	nd	nd	wt#
UT-SCC 42A	larynx	−	mut#	−	nd	nd	wt#
UT-SCC 42B	larynx (m)	−	nd	−	nd	nd	wt#
UT-SCC 60A	tonsil	−	mut#	−	nd	nd	wt#
Cal33	tongue	−	mut**	−	wt**	−**	wt#
HSC4	tongue	−	mut**	−	wt**	−**	wt#
FaDu	hypopharynx	−	mut*	−	wt**	−**	wt#
SAS	tongue	−	mut**	−	wt**	−**	wt#
SAT	oral cavity	−	nd	−	wt**	+**	wt#
XF354	? (m)	−	mut**	−	wt**	−**	wt#

+ positive; - negative; m metastasis; wt wild type; mut mutated; nd not done

* Eicheler et al. 2002 [16]

** Kasten-Pisula et al. 2011 [17];

# own seuqenzing data

### Effect of EGFR inhibition on EGFR signalling and cell proliferation

**Figure 2:**
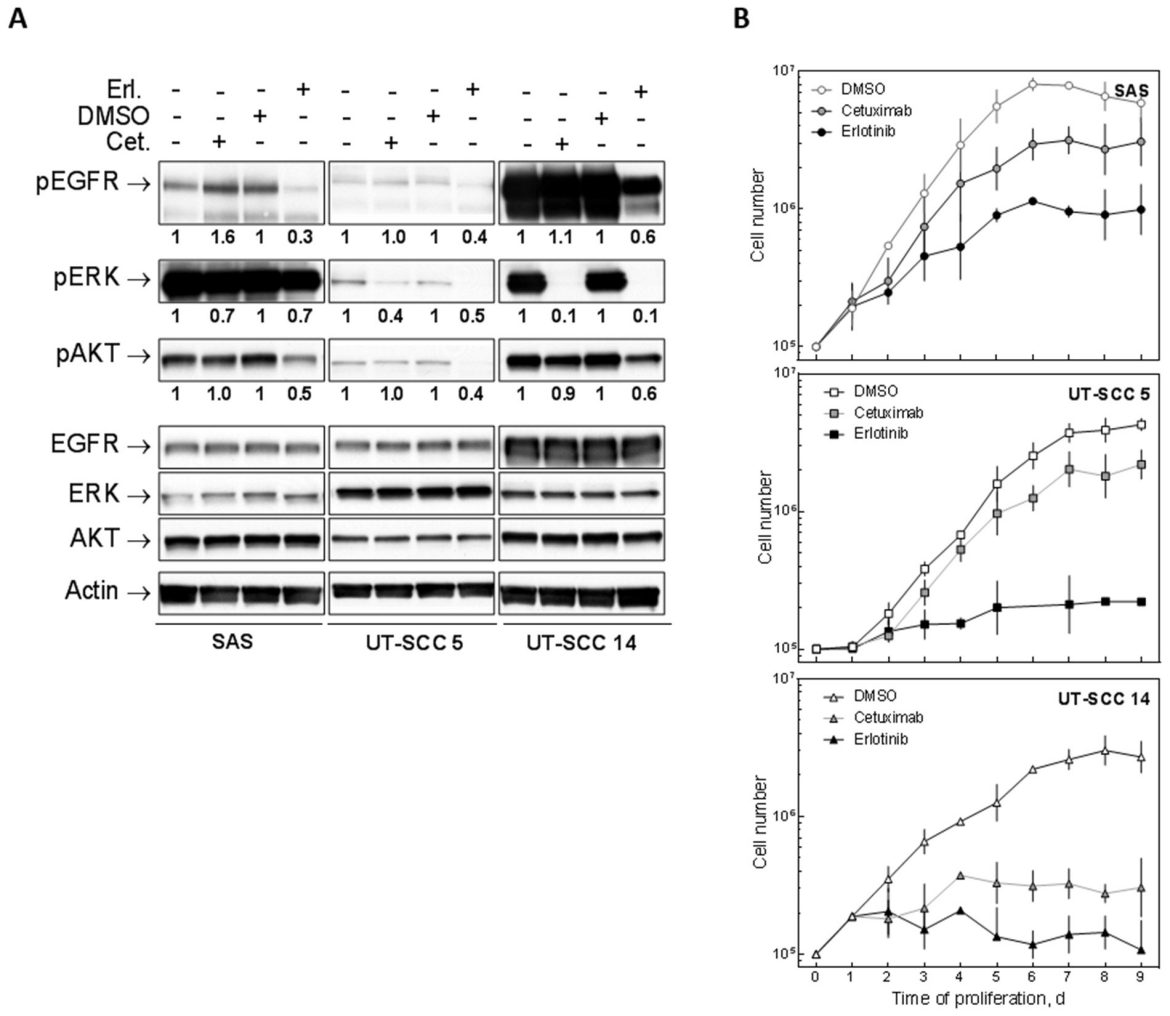
Effect of EGFR inhibition on HNSCC cells SAS, UT-SCC 5 and UT-SCC 14 cells were treated with 5 μM erlotinib or 30 nM cetuximab as indicated. **A.** Signaling: Phosphorylation of EGFR, ERK and AKT was determined by Western blotting after 2 h of treatment. The relative signal intensities are depicted under the corresponding lane. The values of the phospho-signals were normalized to the values of the corresponding unphosphorylated proteins. Cetuximab-treated samples were normalized to untreated ones and erlotinib-treated samples to DMSO-treated ones. **B.** Cell proliferation: The cells were harvested and counted at the indicated time points.

To test whether EGFR inhibition by erlotinib and cetuximab blocks EGFR signaling we detected the phosphorylation of EGFR, ERK and AKT in three cell lines with either low (SAS), intermediate (UT-SCC 5) or very high EGFR expression due to *egfr* gene amplification (UT-SCC 14) by Western blot. We chose 5 μM erlotinib and 30 nM cetuximab since these concentrations already induced maximal proliferation inhibition (Supplementary Figure 1). In line with the strong EGFR expression UT-SCC 14 cells also displayed strong EGFR, ERK and AKT phosphorylation which was blocked by erlotinib (Figure 2A). In contrast, cetuximab only blocked ERK phosphorylation. This was also observed for SAS and UT-SCC 5 cells with SAS displaying even more phospho-EGFR after 2 h cetuximab treatment. Erlotinib also blocked EGFR, ERK and AKT phosphorylation in SAS and UT-SCC 5 cells. The merely moderate inhibition of ERK phosphorylation in SAS in response to erlotinib and cetuximab can be explained by a downstream activation of the MAPK pathway due to Ras overexpression and hyper-activation [16]. Additionally we tested the effect of EGFR inhibition on cell proliferation since a block in proliferation would falsify the analysis of cellular radiosensitivity. Both drugs induced a block in proliferation, with erlotinib causing again a stronger reduction compared to cetuximab and SAS being most resistant while UT-SCC 14 cells, which harbour an *egfr* gene amplification, were most sensitive (Figure 2B). Because of these blocks in proliferation we removed the drugs 24 h after IR in the subsequent colony formation experiments, which restored cell proliferation (data not shown).

### Influence of EGFR inhibition on radiosensitivity under pre- and delayed plating conditions

**Figure 3:**
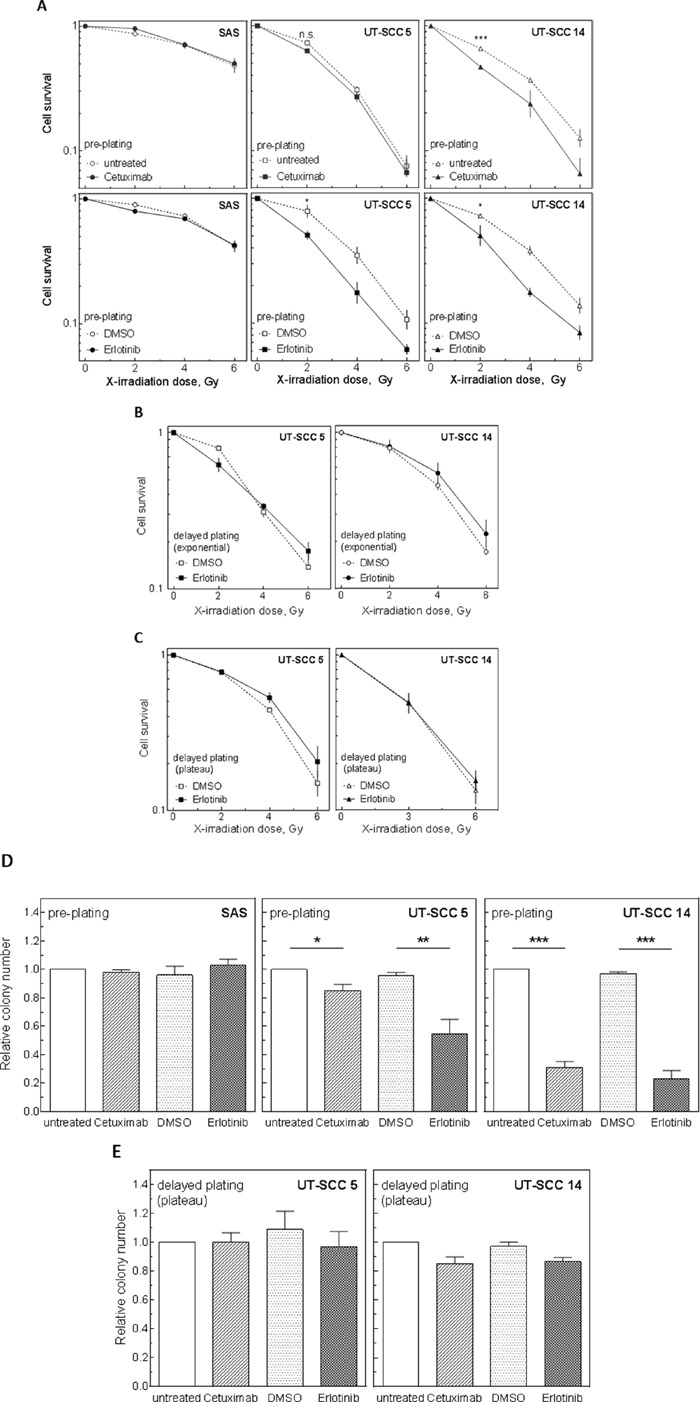
Influence of EGFR inhibition on radiosensitivity and cell survival under pre- and delayed plating conditions SAS, UT-SCC 5 and UT-SCC 14 cells were treated with 5 μM erlotinib or 30 nM cetuximab as indicated. **A-C.** Cells were irradiated with different doses 2 h later. Cell survival measured under (A) pre-plating conditions of exponentially growing cells (inhibitors were removed 24 h after IR, no re-seeding) or (B, C) delayed plating conditions (cells were re-seeded 24 h after irradiation) of (B) exponentially growing cells or (C) plateau phase cells. **D, E.** Cell inactivation by EGFR inhibition alone under (D) pre-plating and (E) delayed plating conditions (plateau phase).

To test radiosensitization by EGFR inhibition in the colony forming assay, cells were treated with erlotinib or cetuximab 2 h before IR and drugs were removed 24 h later. Under pre-plating conditions cetuximab induced radiosensitization only in UT-SCC 14 cells while erlotinib induced a clear sensitization in UT-SCC 5 and UT-SCC 14 cells (Figure 3A). All three sensitizations were found to be significant for 2 Gy. No sensitization was observed for SAS cells.

Strikingly, when the UT-SCC 5 or UT-SCC 14 cells were re-plated 24 h after IR (delayed plating), no sensitization upon EGFR targeting was observable for either exponentially growing (Figure 3B) or plateau phase cells (Figure 3C; Supplementary Figure 2A). Even extending the time of treatment up to 24 h did not provoke any radiosensitization under delayed plating conditions (Supplementary Figure 2B).

Like the radiosensitization also the effect of erlotinib and cetuximab on cell inactivation was dependent on the plating conditions: under pre-plating conditions both drugs caused a significant reduction in the plating efficiency of UT-SCC 5 and UT-SCC 14 cells, with erlotinib causing a stronger reduction (Figure 3D) while under delayed plating conditions this reduction was abolished (Figure 3E). For DMSO-treated samples the absolute plating efficiency was not altered much by changing the plating condition, indicating no selection bias caused by re-plating (Supplementary Figure 3).

**Figure 4:**
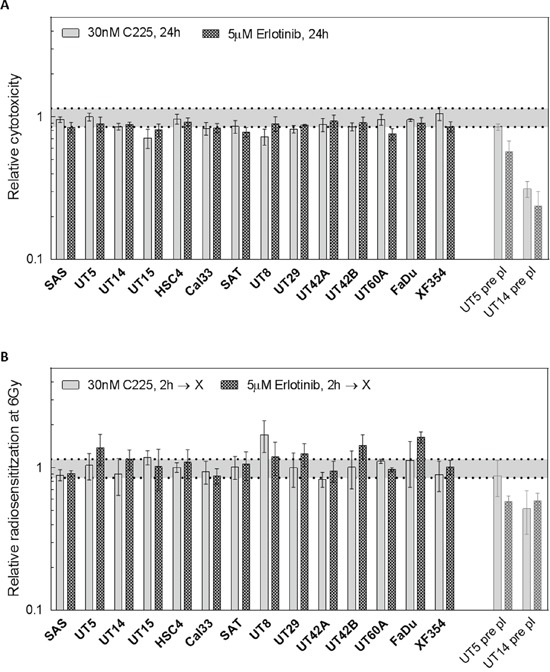
Radiosensitivity after EGFR inhibition in 14 HNSCC cell lines under delayed plating conditions Confluent cultures of 14 different HNSCC cell lines were treated with 5 μM erlotinib or 30 nM cetuximab for 2 h. Cells were irradiated 2 h later with different doses as indicated and were re-plated 24 h later. The data for SAS, UT-SCC 5 and UT-SCC 14 cells were already depicted in Figure 3, and pre-plating data were included (pale bars). **A.** Cytotoxicity: Relative effect of erlotinib and cetuximab on colony formation without IR. **B.** Radiosensitivity: Relative effect of EGFR inhibition on the surviving fraction after 6 Gy of IR (SF6).

The results presented so far indicate that EGFR targeting does not cause a robust radiosensitization. To verify this result in a large cohort of HPV-negative HNSCC cell lines, we tested the other 11 cell lines including 9 cell lines derived from primary tumors and 2 from metastases (Table 1, Supplementary Figure 4). Since KRAS status has been reported to be of importance for EGFR mediated radiosensitization [17, 18] we also sequenced KRAS exons 2-4 in all cell lines detecting only wt sequences (Table 1). In respect to cytotoxicity we observed, on average, only a moderate reduction after erlotinib or cetuximab treatment without IR using plateau phase cells and delayed plating conditions (Figure 4A). In combination with IR no significant increase in cellular radiosensitivity was observed for any cell line under the same conditions (Figure 4B).

### Influence of EGFR inhibition on apoptosis and DNA repair foci

**Figure 5:**
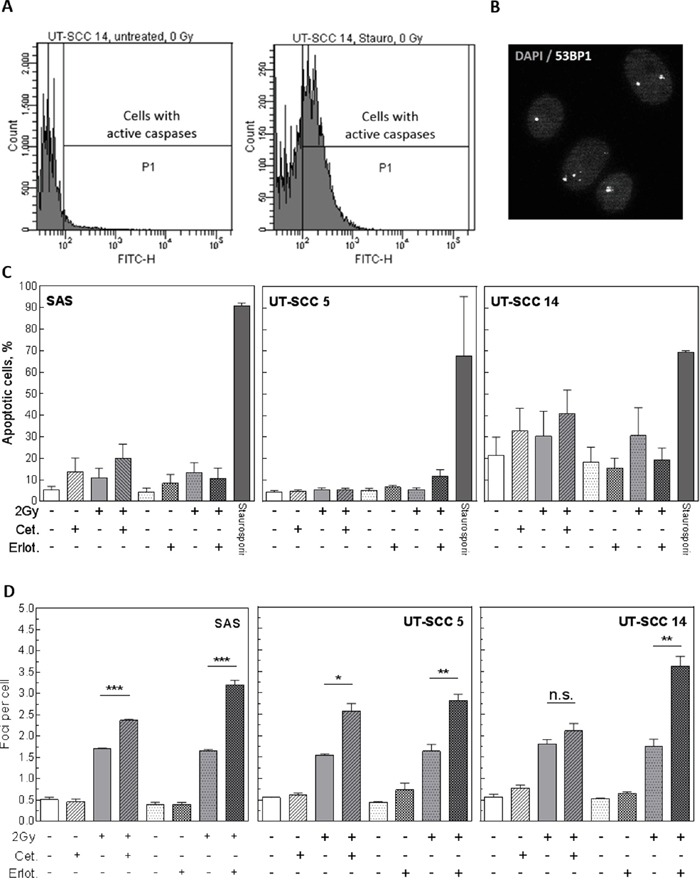
Influence of EGFR inhibition on apoptosis and DNA repair Exponentially growing SAS, UT-SCC 5 and UT-SCC 14 cells were treated with 5 μM erlotinib or 30 nM cetuximab as indicated. Cells were irradiated with different doses 2 h later. **A, C.** Apoptosis: Twenty-four hours after IR cells were fixed and analyzed for primary apoptosis by staining of activated caspases and subsequent flow cytometry. Cells treated with 1 μM staurosporine served as a positive control. (A) Exemplary histograms for untreated and staurosporine treated UT-SCC 14 cells (X-axis: caspase activity). (C) Quantification. **B, D.** DSB repair: To determine DSB repair residual DNA DSB were stained and quantified by immunofluorescence using antibodies against 53BP1 protein. (B) Exemplary picture of residual 53BP1 (white) foci 24 h after 2 Gy in UT-SCC 5 cells. The DNA was stained with DAPI (grey). (D) Quantification.

The data presented so far indicate that radiosensitization of HNSCC cells only occurs under pre-plating conditions but is abolished after re-plating (delayed plating). To analyse which mechanisms are involved in the sensitization observed under pre-plating, we first analysed the induction of apoptosis by flow cytometry (Figure 5A) and the repair of DNA double strand breaks (DSB) by detecting residual 53BP1-positive repair foci via immunofluorescence microscopy (Figure 5B). For SAS, UT-SCC 5 and UT-SCC 14 cells no increase in the fraction of apoptotic cells could be observed 24 h after IR in the samples treated with EGFR inhibitors and IR compared to the irradiated only samples (Figure 5C). In contrast, an increased number of residual DSB was detected after IR in almost all EGFR inhibitor-treated samples (Figure 5D). As this was observed also for SAS cells and for cetuximab-treated UT-SCC 5 cells, the increase in residual DSB did not correlate with radiosensitization under pre-plating conditions.

### Influence of EGFR inhibition on the cell cycle distribution

**Figure 6:**
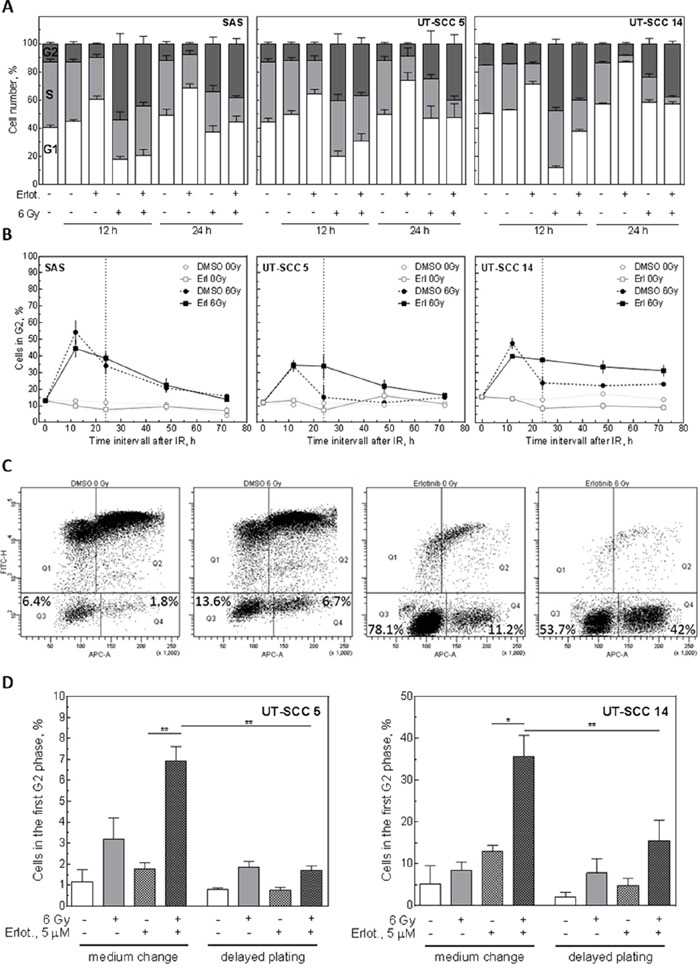
Influence of EGFR inhibition on the cell cycle Exponentially growing SAS, UT-SCC 5 and UT-SCC 14 cells were treated with 5 μM erlotinib or 30 nM cetuximab 2 h before to IR. **A.** Cell cycle distribution 12 h and 24 h after IR as determined by PI staining and flow cytometry. **B.** Kinetics of G2 phase. Medium was changed 24 h after IR (dotted line). Values for 12 and 24 h were taken from A. **C, D.** Determination of G2-arrested cells using EdU incorporation and PI staining analyzed by flow cytometry. EdU was given 2 h after medium change (pre-plating conditions without re-seeding) or re-plating (delayed plating conditions with re-seeding 24 h after irradiation), respectively. (C) Exemplary measurement of UT-SCC 14 cells 24 h after medium change. (D) G2 population 72 h after EdU administration.

The previous experiments have demonstrated, that radiosensitization does not correlate with the induction of apoptosis or residual DSB. Because we have recently demonstrated, that radiosensitization can be associated with the induction of distinct cell cycle arrests [10], we asked whether an EGFR-mediated cell cycle block might correlate with radiosensitization also in this study. To this end, we analysed the cell cycle distribution after IR in combination with erlotinib treatment. While erlotinib alone caused an accumulation of cells in the G1-phase of the cell cycle, IR induced an accumulation in S/G2, as expected due to the induction of DNA damage (Figure 6A). Treatment with erlotinib 2 h before IR reduced the number of S/G2 cells 12 h after IR. However, 24 h after IR more G2 cells were detectable in the erlotinib/IR treated samples compared to the samples treated with IR alone. This was especially pronounced for the UT-SCC 5 and UT-SCC 14 cells (Figure 6A). To investigate the transition through G2 in more detail we removed erlotinib 24 h after IR and analysed the G2 population up to 72 h after IR. These analyses revealed a strongly delayed G2 efflux in double-treated UT-SCC 5 and UT-SCC 14 cells while no such effect was observed for SAS cells (Figure 6B).

To determine if this G2 block can be abolished by re-plating, we used EdU incorporation. As depicted in Figure 6C, EdU staining of UT-SCC 14 cells revealed that nearly all untreated cells migrated through the cell cycle within 24 h. In contrast, when cells were treated with IR or erlotinib 24 h before adding Edu (erlotinib was removed 2 h before adding EdU), a fraction of cells stayed in G1 and G2. Strikingly, following combined treatment, the fraction of G2-arrested cells increased dramatically. Such an increase was not detectable for G1-arrested cells (Figure 6C, Supplementary Figure 5). Quantifying the amount of G2-arrested cells 48 h after adding EdU, we detected a significant increase in the erlotinib- plus IR-treated samples compared to the erlotinib-only-treated samples for UT-SCC 5 and UT-SCC 14 cells (Figure 6D). However, when the cells were re-stimulated 24 h after IR by re-plating, this strong arrest in G2 was significantly reduced.

In summary, these data strongly indicate that radiosensitization observed under pre-plating conditions depends on a reversible arrest of erlotinib- and IR-treated cells in G2.

## DISCUSSION

Using a large panel of 14 independent HPV-negative HNSCC cell lines we clearly demonstrate in this study that targeting the EGFR fails to cause a robust cellular radiosensitization. Whilst cellular radiosensitization can be observed in some cell lines under pre-plating conditions, re-stimulation of the cells by re-plating abolished the sensitization. This effect was recently described by us also for glioblastoma [19] and NSCLC cell lines [10]. In the latter study we also observed no improved tumor control for NSCLC xenografts treated with fractionated IR and EGFR inhibitors erlotinib or cetuximab [10]. For some HNSCC cells, including a few of the cells tested in the present study, Gurtner et al. reported improved tumor control only after cetuximab treatment but not after erlotinib treatment in combination with fractionated IR [20]. Since erlotinib always causes stronger biological effects compared to cetuximab we assume that improved tumor control by cetuximab may not be caused by cellular radiosensitization but rather by a residual immune response in the NMRI (nu/nu) mice, especially for tumors with strong EGFR expression such as UT-SCC 14-derived tumors [20, 21]. This is in line with both the positive results from Bonner et al. reporting better survival after combining cetuximab with RT [1] and the failure of recent clinical trials combining either fully humanized anti-EGFR antibodies or small molecule inhibitors with radiotherapy and radio-chemotherapy [22–24]. Why also cetuximab failed to improve radio-chemotherapy has to be examined in the future [25]. Putting all these findings together, the doctrine that EGFR targeting radiosensitizes HNSCC cells, which accounts for improved patient survival, has to be reconsidered.

Like for NSCLC cell lines radiosensitization of UT-SCC 5 and UT-SCC 14 cells under pre-plating conditions seems to depend on the induction of a reversible cell cycle block [10]. In contrast to p53 wt NSCLC cells, the p53/p21 signaling-deficient HNSCC cells did not arrest in G1 (Supplementary Figure 5). Instead, they showed a pronounced G2-phase arrest which was associated with radiosensitization. Therefore we assume that a lasting G2 arrest is responsible for the radiosensitization observed in p53 mutated cells since it was abolished by re-plating which also abolished radiosensitization. To our knowledge, this is the first study proposing such a mechanism of radiosensitization in HNSCC cells. The failure of erlotinib to improve tumor control in the animal studies [20] proves that this cell cycle arrest-dependent radiosensitization does not translate into improved tumor control and is therefore unlikely to contribute to a clinical effect of EGFR targeting in HNSCC patients. This is again in line with the data obtained for NSCLC cell lines and xenografts [10].

Under pre-plating conditions the putative radiosensitization as well as the inhibition of proliferation and the reduction of clonogenicity by EGFR targeting alone (plating efficiency) seem to correlate with the EGFR expression (SAS < UT-SCC 5 < UT-SCC 14). But again, the strong reduction in the plating efficiency under pre-plating conditions (Figure 3D) can also be attributed to a cell cycle blockage because it is resolved by re-plating. In that case the arrest of cells in G1 seems to be of relevance (Supplementary Figure 5). Still, even under delayed plating conditions, some cell lines showed a moderate reduction in clonogenicity which does not correlate with EGFR expression level (Figure 4A). The factors causing the variations in cell inactivation between the different cell lines are not clear so far.

We have recently published that EGFR targeting inhibits DNA DSB repair in HNSCC cells via MAPK signalling and PARP1 [26]. In this study we also observed elevated residual 53BP1 foci, indicative of an inhibition of DNA DSB repair (Figure 5B). However, it does not correlate with cellular radiosensitization since an increased amount of residual 53BP1 was detected also in SAS cells, which do not become sensitized. Additionally, using delayed plating conditions, an increased number of foci was detected in UT-SCC 5 and SAS cells, too [26]. Whilst the quantification of residual DSB repair complexes using marker proteins such as 53PB1 is a very well established method, further endeavours have to be made to answer why residual repair foci do not correlate with cellular survival in the context of combined EGFR targeting and IR, a phenomenon which has been described already by other studies [10, 27].

In addition to the importance of this study for the understanding of radiosensitization by EGFR targeting, its findings may apply more generally to targeting strategies using kinase inhibitors to induce radiosensitization in cell culture: Firstly our results highlight the importance of choosing the adequate experimental setup (pre- vs. delayed plating) and secondly they broaden the portfolio of potential mechanisms causing reduced colony formation (a persistent G2 arrest). Yet, whether re-plating can be assumed to be more relevant for the *in vivo* situation is hard to say. In our view, both techniques, pre- and delayed plating may reflect different *in vivo* scenarios: whilst pre-plating might better reflect xenograft experiments using irradiation with a single dose or a few fractions, re-plating might better reflect the situation of normal fractionation where repopulation and therefore re-stimulating events take place throughout the course of treatment [12]. This assumption fits quite well to the published xenograft data: whilst effects of single dose irradiation or irradiation with up to five fractions show an additional benefit of targeting EGFR in combination with irradiation [28, 29], fractionated irradiation in combination with EGFR-inhibition might not [20]. However, these considerations are impeded by the fact, that cetuximab might improve the outcome in xenograft experiments by mediating different kinds of immune responses, as discussed above.

In summary we have shown, that radiosensitization of p53/p21-deficient HPV-negative HNSCC cells can occur but only under pre-plating conditions. We also demonstrate here for the first time that this sensitization seems to depend on the arrest of the cells in the G2 phase of the cell cycle and could therefore be abolished by re-plating (re-stimulation), a method which also abolishes the EGFR-dependent G2 cell cycle arrest. Together with our own data showing also no radiosensitization by cetuximab in a panel of 5 HPV-positive HNSCC cell lines [30] and in agreement with other preclinical and recent clinical studies we conclude that EGFR inhibition is no effective strategy to increase the radiosensitivity of HNSCC cells.

## MATERIALS AND METHODS

### Cell lines

HPV-negative HNSCC and A549 (NSCLC) cells were grown in D-MEM medium (Invitrogen) containing 10% FCS (PAN Biotech) and 2 mM glutamine (Invitrogen) at 37°C and 100% humidification. Cells were identified by a short tandem repeat multiplex assay (Applied Biosystems) if a reference was available. Cell lines UT-SCC-8, UT-SCC-14 and SAT harbour *egfr* gene amplifications (Table 1).

### Substances

Erlotinib (Tarceva^®^; Roche), cetuximab (Erbitux^®^; Merck), staurosporine (Sigma-Aldrich) DMSO (vehicle; Roche), propidium iodide (Merck), RNase A (Serva).

### Irradiation (IR)

Cells were irradiated at room temperature with 200 kV X-rays (Gulmay RS225, Gulmay Medical Ltd.; 15 mA, 0.8 mm Be + 0.5 mm Cu filtering; dose rate of 1.2 Gy/min).

### Western blotting

Proteins from whole cell extracts were detected by Western blot according to standard protocols. Primary antibodies: anti-EGFR, anti-pEGFR, anti-ERK1/2, anti-pERK, anti-AKT and anti-pAKT (Cell Signaling Technology), anti-p53 (Novocastra), anti-p21 (Pharmingen), anti-actin (Sigma-Aldrich). Secondary antibodies: anti-mouse and anti-rabbit antibodies (LI-COR Biosciences). The Odyssey^®^ CLx Infrared Imaging System (LI-COR Biosciences) was used for signal detection. Relative signal intensities were given as the quotient of [phospho-protein / unphosphorylated proteins]. Cetuximab-treated samples were normalized to untreated ones and erlotinib-treated samples to DMSO-treated ones.

### Cell proliferation and survival

To measure proliferation, cells were seeded, treated with EGFR-inhibitors 24 h later and cell numbers were determined at the indicated time points. Cell survival was measured by colony formation either under pre-plating or delayed plating conditions. For pre-plating experiments the cells were seeded 24 h before inhibitor treatment. After 2 h the cells were irradiated and the medium was changed 24 h later, keeping the cells without inhibitor for the rest of the experiment. For delayed plating experiments the cells were treated as described above but were trypsinized and re-seeded 24 h after IR (re-plating), inducing a re-stimulation. Cells were grown without inhibitors until colonies reached equal size. Colonies were fixed, stained with crystal violet and the colonies of more than 50 cells were scored as ‘survivors’. The surviving fraction was normalized to the plating efficiency of the non-irradiated controls.

### Cell cycle analysis

#### DNA content

At different time intervals after IR cells were harvested and fixed with ethanol, washed with PBS (0.1% Tween) and stained with propidium iodide solution (10 μg/ml, RNase A 0.1 μg/ml) for 30 min at room temperature. DNA histograms as obtained by flow cytometry (FACS Scan Canto and FACSDiva software, BD Biosciences) were used to determine the fraction of G1-, G2- and S-phase cells using ModFit LTTM software (Verity Software House, Inc.).

#### EdU-incorporation

Twenty-four hours after IR the medium of the cells was either changed or the cells were re-plated. Two hours later the nucleoside analog 5-ethynyl-2′-deoxyuridine (EdU) was added. Cells were fixed at different time points as indicated, stained for EdU according to the manufacturer's protocol (Baseclick) and stained with propidium iodide as described above. DNA histograms and EdU incorporation were analysed using FACS Scan Canto and FACSDiva software (BD Biosciences).

### Immunofluorescence / DNA repair

Residual DNA repair foci were analyzed by immunofluorescence staining as described earlier [31]. Briefly, cells were fixed and stained with anti-53BP1 (Novus, Biologicals) antibodies followed by fluorescein-labeled anti-rabbit (GE-Healthcare, Amersham™) secondary antibodies. DNA was stained with 4′,6-diamidino-2-phenylindole (DAPI; QBiogene). A confocal fluorescence microscope (Zeiss Axioplan 2; 630-fold magnification) was used for analysis of 53BP1 foci. At least 100 nuclei were randomly selected and foci were counted by eye. Only intact nuclei were analyzed.

### Apoptosis

For the detection of apoptosis, cells were analyzed 24 h after IR by measuring caspase activity employing flow cytometry and the *Carboxyfluorescin FLICA Apoptosis Detection Kit Caspase Assay* (Immunochemistry Technologies, LLC), according to the manufacturer's protocol.

### KRAS sequencing

Sequencing of KRAS exons 2/3/4 was performed as described previously [32].

### Data evaluation

Unless otherwise indicated, experiments were repeated at least three times. The data are presented as mean values (±SEM). Prism 5 software (GraphPad Software) was used for analyzing and graphing the data. The unpaired student's t-test was performed for the statistical analysis. P-values were calculated using two-sided tests (*p < 0.05; **p < 0.01; ***p < 0.001).

## SUPPLEMENTARY FIGURES



## References

[1] Bonner JA, Harari PM, Giralt J, Cohen RB, Jones CU, Sur RK, Raben D, Baselga J, Spencer SA, Zhu J, Youssoufian H, Rowinsky EK, Ang KK (2010). Radiotherapy plus cetuximab for locoregionally advanced head and neck cancer: 5-year survival data from a phase 3 randomised trial, and relation between cetuximab-induced rash and survival. Lancet Oncol.

[2] Baumann M, Krause M, Dikomey E, Dittmann K, Dorr W, Kasten-Pisula U, Rodemann HP (2007). EGFR-targeted anti-cancer drugs in radiotherapy: preclinical evaluation of mechanisms. Radiother Oncol.

[3] Dittmann K, Mayer C, Rodemann HP (2005). Inhibition of radiation-induced EGFR nuclear import by C225 (Cetuximab) suppresses DNA-PK activity. Radiother Oncol.

[4] Tanaka T, Munshi A, Brooks C, Liu J, Hobbs ML, Meyn RE (2008). Gefitinib radiosensitizes non-small cell lung cancer cells by suppressing cellular DNA repair capacity. Clin Cancer Res.

[5] Toulany M, Kasten-Pisula U, Brammer I, Wang S, Chen J, Dittmann K, Baumann M, Dikomey E, Rodemann HP (2006). Blockage of epidermal growth factor receptor-phosphatidylinositol 3-kinase-AKT signaling increases radiosensitivity of K-RAS mutated human tumor cells in vitro by affecting DNA repair. Clin Cancer Res.

[6] Chinnaiyan P, Huang S, Vallabhaneni G, Armstrong E, Varambally S, Tomlins SA, Chinnaiyan AM, Harari PM (2005). Mechanisms of enhanced radiation response following epidermal growth factor receptor signaling inhibition by erlotinib (Tarceva). Cancer Res.

[7] Zhuang HQ, Sun J, Yuan ZY, Wang J, Zhao LJ, Wang P, Ren XB, Wang CL (2009). Radiosensitizing effects of gefitinib at different administration times in vitro. Cancer science.

[8] Giocanti N, Hennequin C, Rouillard D, Defrance R, Favaudon V (2004). Additive interaction of gefitinib (‘Iressa’, ZD1839) and ionising radiation in human tumour cells in vitro. Br J Cancer.

[9] Chang GC, Hsu SL, Tsai JR, Liang FP, Lin SY, Sheu GT, Chen CY (2004). Molecular mechanisms of ZD1839-induced G1-cell cycle arrest and apoptosis in human lung adenocarcinoma A549 cells. Biochemical pharmacology.

[10] Kriegs M, Gurtner K, Can Y, Brammer I, Rieckmann T, Oertel R, Wysocki M, Dorniok F, Gal A, Grob TJ, Laban S, Kasten-Pisula U, Petersen C, Baumann M, Krause M, Dikomey E (2015). Radiosensitization of NSCLC cells by EGFR inhibition is the result of an enhanced p53-dependent G1 arrest. Radiother Oncol.

[11] Wang M, Morsbach F, Sander D, Gheorghiu L, Nanda A, Benes C, Kriegs M, Krause M, Dikomey E, Baumann M, Dahm-Daphi J, Settleman J, Willers H (2011). EGF receptor inhibition radiosensitizes NSCLC cells by inducing senescence in cells sustaining DNA double-strand breaks. Cancer Res.

[12] Baumann M, Krause M (2004). Targeting the epidermal growth factor receptor in radiotherapy: radiobiological mechanisms, preclinical and clinical results. Radiother Oncol.

[13] Withers HR, Peters LJ, Taylor JM, Owen JB, Morrison WH, Schultheiss TE, Keane T, O'Sullivan B, van Dyk J, Gupta N (1995). Local control of carcinoma of the tonsil by radiation therapy: an analysis of patterns of fractionation in nine institutions. International journal of radiation oncology, biology, physics.

[14] Eicheler W, Zips D, Dorfler A, Grenman R, Baumann M (2002). Splicing mutations in TP53 in human squamous cell carcinoma lines influence immunohistochemical detection. J Histochem Cytochem.

[15] Kasten-Pisula U, Saker J, Eicheler W, Krause M, Yaromina A, Meyer-Staeckling S, Scherkl B, Kriegs M, Brandt B, Grenman R, Petersen C, Baumann M, Dikomey E (2011). Cellular and Tumor Radiosensitivity is Correlated to Epidermal Growth Factor Receptor Protein Expression Level in Tumors Without EGFR Amplification. International journal of radiation oncology, biology, physics.

[16] Saki M, Toulany M, Rodemann HP (2013). Acquired resistance to cetuximab is associated with the overexpression of Ras family members and the loss of radiosensitization in head and neck cancer cells. Radiother Oncol.

[17] Toulany M, Dittmann K, Kruger M, Baumann M, Rodemann HP (2005). Radioresistance of K-Ras mutated human tumor cells is mediated through EGFR-dependent activation of PI3K-AKT pathway. Radiother Oncol.

[18] Toulany M, Dittmann K, Baumann M, Rodemann HP (2005). Radiosensitization of Ras-mutated human tumor cells in vitro by the specific EGF receptor antagonist BIBX1382BS. Radiother Oncol.

[19] Struve N, Riedel M, Schulte A, Rieckmann T, Grob TJ, Gal A, Rothkamm K, Lamszus K, Petersen C, Dikomey E, Kriegs M (2015). EGFRvIII does not affect radiosensitivity with or without gefitinib treatment in glioblastoma cells. Oncotarget.

[20] Gurtner K, Deuse Y, Butof R, Schaal K, Eicheler W, Oertel R, Grenman R, Thames H, Yaromina A, Baumann M, Krause M (2011). Diverse effects of combined radiotherapy and EGFR inhibition with antibodies or TK inhibitors on local tumour control and correlation with EGFR gene expression. Radiother Oncol.

[21] Hsu YF, Ajona D, Corrales L, Lopez-Picazo JM, Gurpide A, Montuenga LM, Pio R (2010). Complement activation mediates cetuximab inhibition of non-small cell lung cancer tumor growth in vivo. Molecular cancer.

[22] Eriksen JG, Maare C, Johansen J, Primdahl H, Evensen J, Kristensen CA, Andersen LJ, Overgaard J (2014). DAHANCA19: A randomized phase III study of primary (chemo-) radiotherapy and zalutumumab in head and neck carcinomas. Radiother Oncol.

[23] Martins RG, Parvathaneni U, Bauman JE, Sharma AK, Raez LE, Papagikos MA, Yunus F, Kurland BF, Eaton KD, Liao JJ, Mendez E, Futran N, Wang DX, Chai X, Wallace SG, Austin M (2013). Cisplatin and Radiotherapy With or Without Erlotinib in Locally Advanced Squamous Cell Carcinoma of the Head and Neck: A Randomized Phase II Trial. J Clin Oncol.

[24] Van Waes C, Allen CT, Citrin D, Gius D, Colevas AD, Harold NA, Rudy S, Nottingham L, Muir C, Chen Z, Singh AK, Dancey J, Morris JC (2010). Molecular and clinical responses in a pilot study of gefitinib with paclitaxel and radiation in locally advanced head-and-neck cancer. International journal of radiation oncology, biology, physics.

[25] Ang KK, Zhang Q, Rosenthal DI, Nguyen-Tan PF, Sherman EJ, Weber RS, Galvin JM, Bonner JA, Harris J, El-Naggar AK, Gillison ML, Jordan RC, Konski AA, Thorstad WL, Trotti A, Beitler JJ (2014). Randomized phase III trial of concurrent accelerated radiation plus cisplatin with or without cetuximab for stage III to IV head and neck carcinoma: RTOG 0522. J Clin Oncol.

[26] Myllynen L, Kwiatkowski M, Gleissner L, Riepen B, Hoffer K, Wurlitzer M, Petersen C, Dikomey E, Rothkamm K, Schluter H, Kriegs M (2015). Quantitative proteomics unveiled: Regulation of DNA double strand break repair by EGFR involves PARP1. Radiother Oncol.

[27] Stegeman H, Span PN, Cockx SC, Peters JP, Rijken PF, van der Kogel AJ, Kaanders JH, Bussink J (2013). EGFR-inhibition enhances apoptosis in irradiated human head and neck xenograft tumors independent of effects on DNA repair. Radiation research.

[28] Harari PM, Huang SM (2001). Head and neck cancer as a clinical model for molecular targeting of therapy: combining EGFR blockade with radiation. International journal of radiation oncology, biology, physics.

[29] Stegeman H, Kaanders JH, van der Kogel AJ, Iida M, Wheeler DL, Span PN, Bussink J (2013). Predictive value of hypoxia, proliferation and tyrosine kinase receptors for EGFR-inhibition and radiotherapy sensitivity in head and neck cancer models. Radiother Oncol.

[30] Guster JD, Weissleder SV, Busch CJ, Kriegs M, Petersen C, Knecht R, Dikomey E, Rieckmann T (2014). The inhibition of PARP but not EGFR results in the radiosensitization of HPV/p16-positive HNSCC cell lines. Radiother Oncol.

[31] Myllynen L, Rieckmann T, Dahm-Daphi J, Kasten-Pisula U, Petersen C, Dikomey E, Kriegs M (2011). In tumor cells regulation of DNA double strand break repair through EGF receptor involves both NHEJ and HR and is independent of p53 and K-Ras status. Radiother Oncol.

[32] Braig F, Marz M, Schieferdecker A, Schulte A, Voigt M, Stein A, Grob T, Alawi M, Indenbirken D, Kriegs M, Engel E, Vanhoefer U, Grundhoff A, Loges S, Riecken K, Fehse B (2015). Epidermal growth factor receptor mutation mediates cross-resistance to panitumumab and cetuximab in gastrointestinal cancer. Oncotarget.

