# Supplementary material for: Radiosensitization of HNSCC cells by EGFR inhibition depends on the induction of cell cycle arrests
Source: Oncotarget. 2016 May 4;7(29):45122–33. doi: 10.18632/oncotarget.9161 (PMC5216710; doi:10.18632/oncotarget.9161)
Supplement: Supplementary file 1 [file oncotarget-07-45122-s001.pdf]

Radiosensitization of HNSCC cells by EGFR inhibition depends on the induction of cell cycle arrests

SUPPLEMENTARY FIGURES

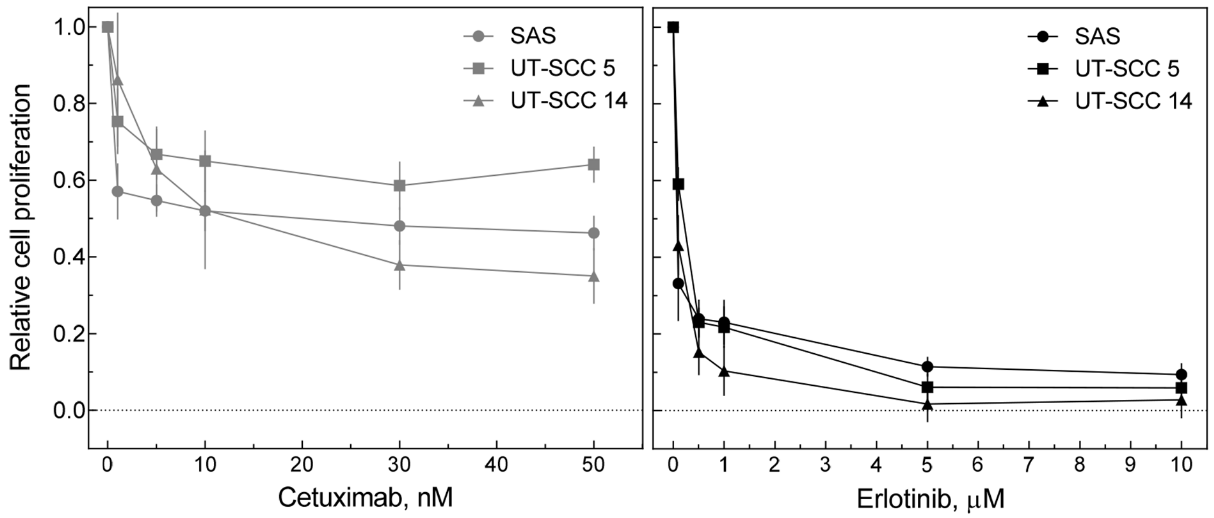

Supplementary Figure S1: EGFR inhibition blocks proliferation. Dose-response of SAS, UT-SCC 5 and UT-SCC 14 cells. Cells were cultivated in the presence of different concentrations erlotinib or cetuximab as indicated. Cell numbers were measured after 5 days of treatment and were normalized to the untreated control.

A)

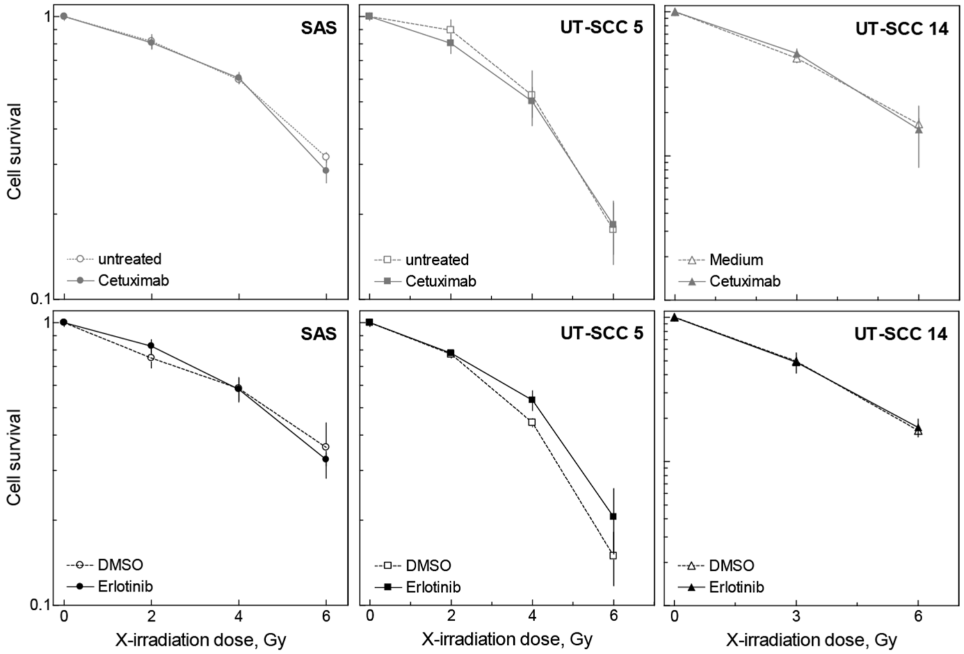

B)

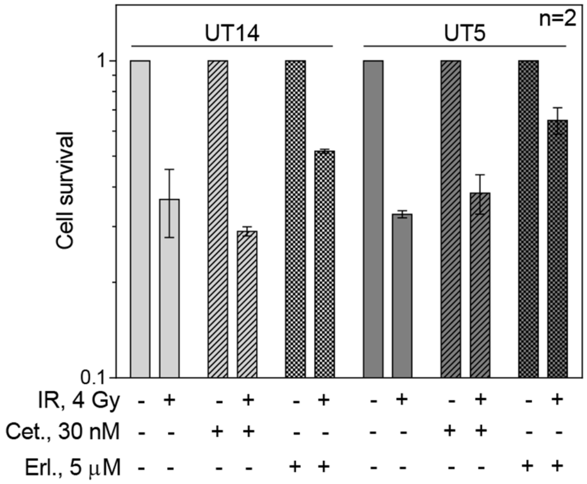

Supplementary Figure S2: Influence of EGFR inhibition on radiosensitivity. A. SAS, UT-SCC 5 and UT-SCC 14 cells were treated with 5 μM erlotinib or 30 nM cetuximab as indicated. Two hours later the cells were irradiated with different doses. Cell survival was measured under delayed plating conditions (cells were re-seeded 24 h after irradiation) in plateau phase cells. The data for erlotinib-treated UT-SCC 5 and UT-SCC 14 cells are also depicted in Figure 3C. **B.** UT-SCC 5 and SAS cells were treated with 5 μM erlotinib or 30 nM cetuximab for 24 h prior to irradiation. Cell survival measured under delayed plating conditions (cells were re-seeded 24 h after irradiation) in plateau phase cells. The surviving fraction at 4 Gy was normalized to the plating efficiency of the non-irradiated control (mean value of 2 independent experiments).

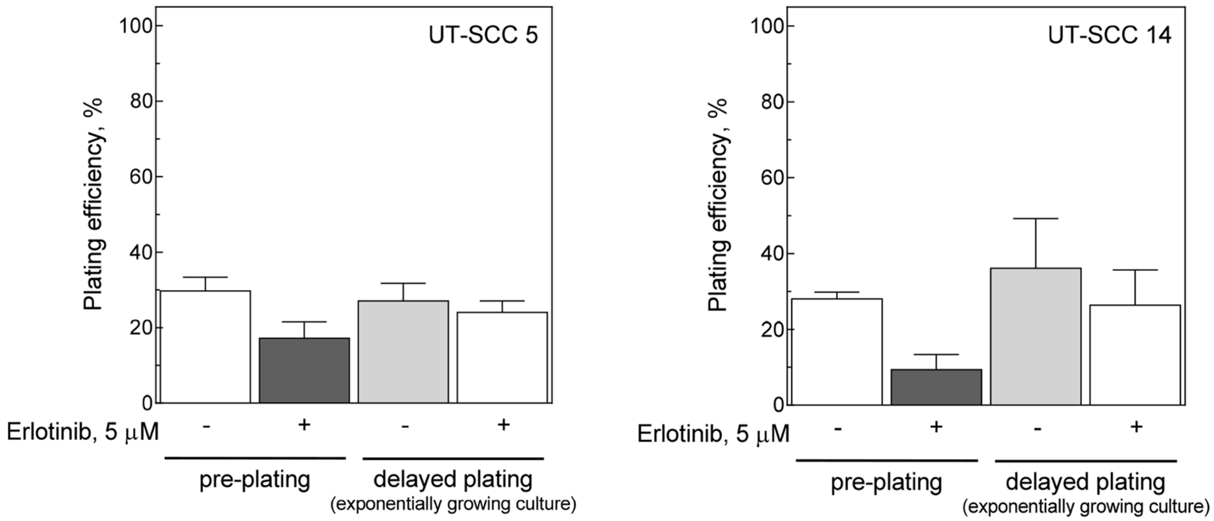

Supplementary Figure S3: Absolute plating efficiencies. Absolut plating efficiencies of UT-SCC 5 and UT-SCC 14 cells treated with DMSO or erlotinib for 24 h. Cells were analysed under either pre-plating (medium exchange after 24 h) or delayed plating conditions (re-plating after 24 h). For delayed plating experiments exponentially growing cells were analyzed.

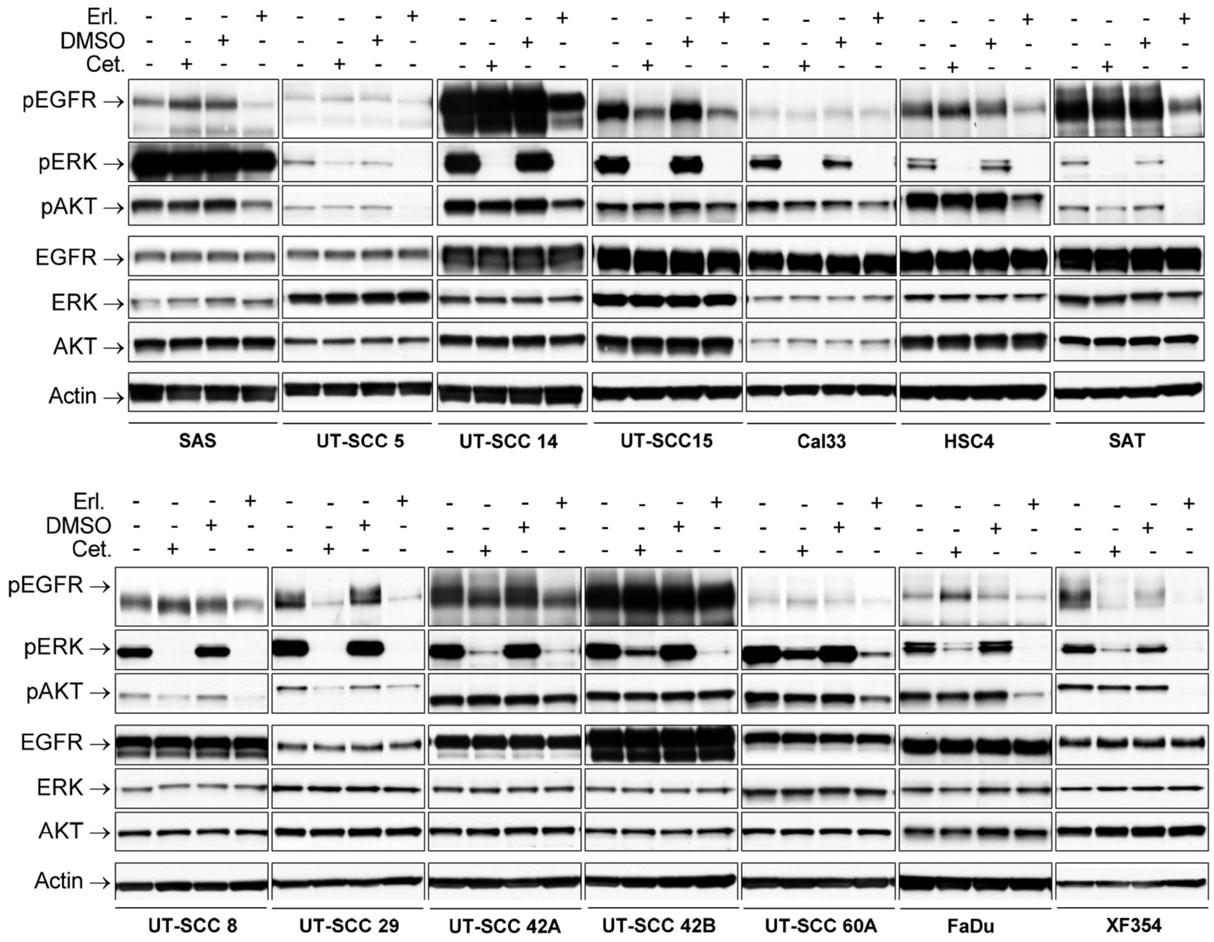

Supplementary Figure S4: Effect of EGFR inhibition on cellular singal transduction. Fouteen different HNSCC cell lines were treated in plateau phase with 5 µM erlotinib or 30 nM cetuximab for 2 h. Cells were lysed and phosphorylation of EGFR, ERK and AKT was analyzed by Western blot using the corresponding antibodies. The blots for SAS, UT-SCC 5 and UT-SCC 14 are also depicted in Figure 2A.

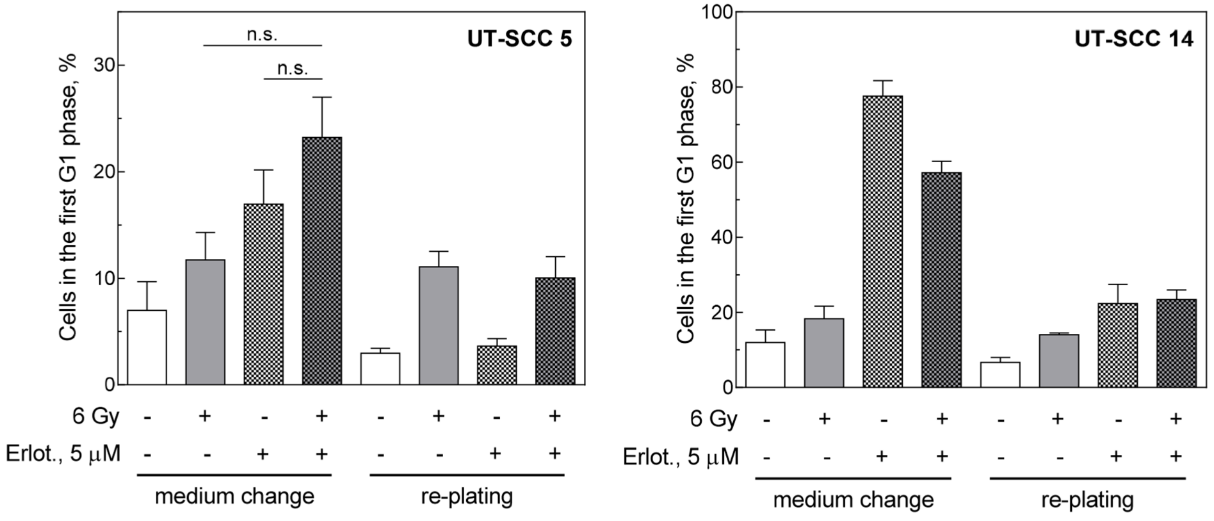

Supplementary Figure S5: G1 arrest. Exponentially growing SAS, UT-SCC 5 and UT-SCC 14 cells were treated with 5 µM erlotinib 2 h prior to IR. Either medium was changed or cells were re-plated 24 h after IR and EdU was given 2 h thereafter. The amount of G1-arrested cells was determined using EdU and PI staining analyzed by flow cytometry 48 h after medium change or re-plating, respectively. Exemplary dot plots for UT-SCC 14 cells are shown in Figure 6C. No additional G1 arrest could be observed in the IR- and erlotinib-treated samples compared to the IR-only-treated samples which might explain the radiosensitization observed in Figure 3A. However, especially for UT-SCC 14 cells, erlotinib induced a strong arrest in G1 under pre-plating conditions independently from IR. This strong G1 arrest was abrogated after re-plating, indicating, that this arrest caused the strong reduction of colony numbers, observed in Figure 3D.
